# Data-driven prediction and thermodynamic performance assessment of industrial cooling towers using advanced machine learning algorithms

**DOI:** 10.1371/journal.pone.0351944

**Published:** 2026-07-02

**Authors:** Syed Rehman Jamil, Adeel Shehzad, Muhammad Usman, Muhammad Mujtaba Abbas, Omama Zainab Qaisrani, Muhammad Wajid Saleem, Hafiz Muhammad Musharaf, Jana Petrů, Muhammad Nasir Bashir, Yasser Fouad

**Affiliations:** 1 Department of Mechanical Engineering, University of Engineering and Technology, Lahore, Pakistan; 2 Automotive Engineering Centre, University of Engineering and Technology, Lahore, Pakistan; 3 Department of Mechanical Engineering, University of Engineering & Technology, New Campus, Lahore, Pakistan; 4 College of Engineering and Energy, Abdullah Al Salem University, Khaldiya, Kuwait; 5 Queensland Quantum and Advanced Technologies Research Institute, Griffith University, Brisbane, Australia; 6 Department of Machining, Assembly and Engineering Metrology, Faculty of Mechanical Engineering, VŠB Technical University of Ostrava, Ostrava, Czech Republic; 7 Multi-Scale Fluid Dynamics Lab, Department of Mechanical Engineering, Yonsei University, Seoul, Republic of Korea; 8 Department of Applied Mechanical Engineering, College of Applied Engineering, Muzahimiyah Branch, King Saud University, Riyadh, Saudi Arabia; Indian Institute of Technology Guwahati, INDIA

## Abstract

Cooling towers are an important part of the thermal system in industries, where they are used to remove unwanted heat and help maintain the proper performance of the machines. Four machine learning algorithms, namely random forest, support vector machine (SVM), decision tree, and AdaBoost are proposed in this paper for the performance forecasting of cooling towers. for the performance forecasting of cooling towers. These models were built in Python with the help of the following operational parameters: inlet water temperature (32–41°C), ambient air temperature (14–32°C), and relative humidity (35–92%). All the essential performance measures like outlet water temperature, water losses, the effectiveness, and the second law efficiency were predicted and assessed by statistical indicators such as coefficient of determination (R²), root mean square error (RMSE) and mean absolute percentage error (MAPE). The SVM algorithm had the best predictive accuracy and lowest prediction errors of all the tested models with a value of R^2^ of 0.985 and RMSE of 1.25 kg/s. Parametric analysis had indicated that the increase in relative humidity between 35% and 92% decreased the evaporation losses by about 55–70% and makeup water demand by about 58–68%. Thermodynamic analysis further revealed that the second-law efficiency improved by approximately 65–75% as the ambient temperature increased. The results indicate that predictive modeling with machine learning offers a useful method in the optimization of cooling tower operation and minimizing water use in industrial systems.

## 1. Introduction

Dissipating excess heat and keeping the system running at the appropriate temperature, cooling towers play an important role in industrial and commercial systems [1–3]. The efficient operation of cooling towers impacts energy consumption, the negative environmental impacts and water usage, and the overall system performance [4–6]. Generally, the current cooling tower control strategies are fixed set points or rule-based methods and usually bring suboptimal energy efficiency and high operational cost [7–9]. Studies further illustrate the intervention difficulties of cooling tower operation and point out the comprehensive as well as individual (wet bulb temperature and thermal inertia) approach on optimization of system overall as requirement and demand machine learning for innovations to cope with the shortcomings.

Lately, advancements in the model predictive control (MPC) and machine learning (ML) have created new opportunities to achieve the highest cooling tower performance by continuously adjusting the key performance parameters, like fan speed, water flow rate, and setpoint temperatures [10,11]. Artificial neural networks (ANN) and support vector regression (SVR) were effectively employed to predict the performance of cooling towers under varying environmental and load conditions [12–14]. Further, MPC techniques using optimisation techniques like genetic algorithms (GA), particle swarm optimisation (PSO) and simulated annealing (SA) have been used to improve the cooling tower performance [15–18]. In recent years fractional calculus has been used in study of heat and mass transfer phenomena in viscoelastic nanofluids with thermal radiation. A number of artificial neural network techniques, especially the Levenberg–Marquardt backpropagation algorithm, was used to increase the accuracy of prediction in complex fluid flow models [19]. Zhong Guo et al. [20] introduced a reinforcement learning controller for chiller and thermal energy storage system operations in district cooling plants, based on Q-learning, which reduced energy costs by almost 8% compared to a conventional rule-based controller. An artificial neural network was created to improve the accuracy of a computer model used to relate the heat discharged to the visible volume of the plume from a 12-cell mechanical draft cooling tower. Three algorithms were applied to 289 cases, with two network configurations enhancing the accuracy of individual predictions to an *R*^2^ > 0.95 level [21]. Nima Assari et al. [12] used optimization algorithms along with artificial neural network (ANN-IOAs) for the optimization of the operational performance of a cooling tower. Two scenarios were examined using input parameters including rotor speed, mass flow rates ratio, and inlet air wet-bulb temperature. The results showed that the BA approach produced the most effective results, with exceptional precision. Avesahemad S. N. Husainy et al. [22] focused on optimizing chiller system performance through machine learning techniques such as regression, classification, or time-series forecasting models, analyzing historical data, and developing predictive models for energy efficiency. Ashish Kumar et al. [23] applied the Genetic Algorithm (GA) and Particle Swarm Optimisation (PSO) to boost the overall performance of cooling tower of steam turbine power plant. The PSO technique was more accurate than GA technique in predicting cooling tower performance. Jiasheng Wu et al. [24] have proposed a cross-flow cooling tower performance parameters prediction model by using back-propagation neural network. The model is able to predict several parameters including the Lewis number, the sensible heat ratio, the heat absorption efficiency, temperature of water, outlet air dry bulb temperature, wet bulb temperature and so on. The Genetic Algorithm Back Propagation (GABP) model was found to be superior to Back Propagation (BP) network model in terms of performance prediction. The GABP-predicted value and the experimental value had mean relative errors (MREs) of 6.57%, 3.17%, 11.50%, 2.27%, 2.66%, and 3.04%, respectively. Ashish Kumar et al. [25] explored four swarm intelligence-based metaheuristic algorithms to optimize cooling tower availability, i.e., the Whale Optimisation Algorithm, the Dragonfly Algorithm, the Grasshopper Optimisation Algorithm, and the Grey Wolf Optimiser. The most effective was the Whale Optimization Algorithm, which gained optimized failure rates, followed by repairs compared to all other metaheuristic algorithms Xavier Lefebvre et al. [26] analyzed how cross-flow microsand filtration systems affect cooling tower performance. They concluded that large amounts of energy could be saved in units utilizing such systems. Overall, these systems achieved a higher coefficient of performance (COP) of 18% and 63% higher when using the filter. According to the model, if operated year round, energy savings could be between 5% and 13% of the energy bill. An approach based on artificial neural networks was developed by Zhe Tian Xu et al. [27] to estimate the capacity of a forced draft wet cooling tower. The input parameters were the flow rate of water in the pipeline and the mass flow-rate of ambient air, and the wet-bulb temperature and inlet water temperature. The procedure’s practicality and efficacy were demonstrated by a 0.9 correlation coefficient for water temperature at the tower outflow using the four-layer back propagation neural network’s (BPNN) predictions. Nadia Nedjah et al. [11] employed multi-objective particle swarm optimization. The focus was on the solutions that benefit the cooling tower in its efficiency without affecting the system power consumption. With an energy efficiency factor of 1.78 and a decrease in tower effectiveness of 5.32%, power savings of 9.48% were achieved. Jung and Jung [28] established the basis for forecasting nuclear power station cooling tower performance based on variables such as absolute humidity, dry, and wet bulb temperature. To validate the framework, the toolkit was utilized from the American Cooling Tower Industry Association. This framework is essential for power plant design because the results align with manufacturers’ cold water temperature on cooling tower performance curves. In their efforts to improve industrial cooling tower management with the main objective of saving energy, R. Selvakumar et al. [29] proposed a new method. In this project, they used Internet of Things technologies with Gradient Boosting to build a system that measures in real time temperature, flow rate, and humidity from the cooling system. Thus, it allows predictive analytics related to system performance and energy consumption. Shajar Abbas et al. [30] worked on fractional-order models of nanofluids to achieve a more effective study of heat and mass transfer in radiation-dominated environment. Research on blood-water based nanofluids has demonstrated improved thermal characteristics because of the better thermal conductivities of carbon nanotubes. The Decision Tree model and four metaheuristic algorithms were used to predict cooling load, with the Decision Tree model and Prairie Dog Optimization DTPD (DT + PDO) model achieving an impressive R^2^ value of 0.995 [31]. Sisavath Xayyasith et al. [32] utilized SVM and Decision Trees for predictive maintenance of a cooling system at the Nam Ngum-1 hydropower plant, utilizing 22 classifier types, including Decision Trees, Discriminant Analysis, SVM, Logistic Regression, KNN, and Ensemble Classification. SVM and Decision Trees were more effective in predicting results. María C. Bueso et al. [33] compared linear regression and an artificial neural network for estimating evaporated water mass in cooling towers. The Multilayer Perceptron model performed 0.76% better than the linear model. Kuljeet Singh and Ranjan Das [34] made an effort to improve the performance and reduced the energy consumption of the cooling tower by using a multi-objective optimisation model based on the Non-dominated Sorting Genetic Algorithm (NSGA-II). The model focuses on five parameters: range, approach, effectiveness, evaporation rate, and energy efficiency, achieving a maximum 25.6% energy saving. M. A. Mujtaba et al. [35] investigated the impact of ambient parameters on cooling tower efficiency, focusing on site selection. Data from a Pakistani power plant was collected, and an Adaboost regressor model was used to quantify the effect of ambient parameters. The study found that relative humidity was the most important factor, contributing 12% to cooling tower efficiency. K Karunamurthy et al. [36] developed the model using Jupyter Notebook to build it in Python, compared it to theoretical and measured values, examined it using common metrics, and used RMSE and R^2^ values to assess its correctness. The R^2^ score of the model was 0.71, and its RMSE value was 0.96, indicating a modest error rate.

In this work, cooling tower performance was evaluated using machine learning models implemented in Python using the TensorFlow platform. The study was aimed at predicting the key output parameters of a cooling tower, namely, the exit temperatures, wet-bulb temperature, blowdown loss, evaporation loss, makeup water, effectiveness and second-law efficiency from the input parameters such as inlet water temperature, ambient air temperature and inlet air humidity, by employing machine learning approaches. The novelty of this research lies in the comparative analysis of multiple machine learning algorithms for predicting and optimizing cooling tower performance using real industrial data. Unlike previous studies that primarily rely on empirical correlations or single-model approaches, this study leverages advanced machine learning techniques (SVM, Random Forest, Decision Tree and AdaBoost) to perform simultaneous prediction of key thermodynamic and operational parameters. The fact that full-scale industrial operational data is used and that detailed parametric and physical analysis is included further makes this work different than the existing studies.

Moreover, the research complements the Sustainable Development Goals (SDGs) especially by optimizing water use in cooling systems; affordable and clean energy by improving energy efficiency; and climate action by reducing the environmental footprint with smart resource management.

## 2. Materials and methods

The performance of the Head Baloki Power Plant’s induced draft wet cooling tower at Pattoki, Pakistan, was evaluated. In the current study, a set of input and performance parameters were selected based on a comprehensive literature review and recommendations from power plant operators. Table 1 shows the real-time data comprised of metrological parameters (ambient temperature, ambient pressure, relative humidity, wet bulb temperature, blowdown loss, evaporation loss, and outlet water temperature) along with the operational parameters of the cooling tower. All essential data was collected for the whole year of 2023, from January to September, because the power plant was only operational during these months, and the rest of the year was spent on facility maintenance. The data was continuous, and it needed to undergo a filtration process to exclude the startup, shutdown, trip, testing, and sensors’ abnormal operating conditions.

**Table 1:** Actual parameters of the whole year of Head Baloki Cooling Tower.

Parameters	Symbols	Units	Maximum	Minimum	Average
Ambient Air Temperature	T_amb_	°C	32	14	26.44
Relative Humidity	Φ_in_	%	92	35	60.62
Pressure	P_amb_	kpa	100.30	96.99	98.27
Inlet Water Temperature	T_in,w_	°C	41	32	36
Outlet Water Temperature	T_out,w_	°C	37.85	24.28	31.28
Wet Bulb Temperature	T_wb_	°C	30.83	7.106	20.80
Mass Flow Rate of Water	ṁ_w_	kg/s	4722.22	4472.13	4597.18
Air Flow Rate	ṁ_a_	kg/s	2773.04	2577.66	2675.35
Blowdown Loss	BL	kg/s	18.41	0.6375	18.61
Evaporation Loss	EL	kg/s	73.66	2.54	74.44
Makeup Water	ṁ_mw_	kg/s	101.53	12.63	102.50
Effectiveness	η	%	32	31	31.02
Second Law Efficiency	η_II_	%	0.93	0.15	0.96

### 2.1. Sequence of analysis

**Fig 1:**
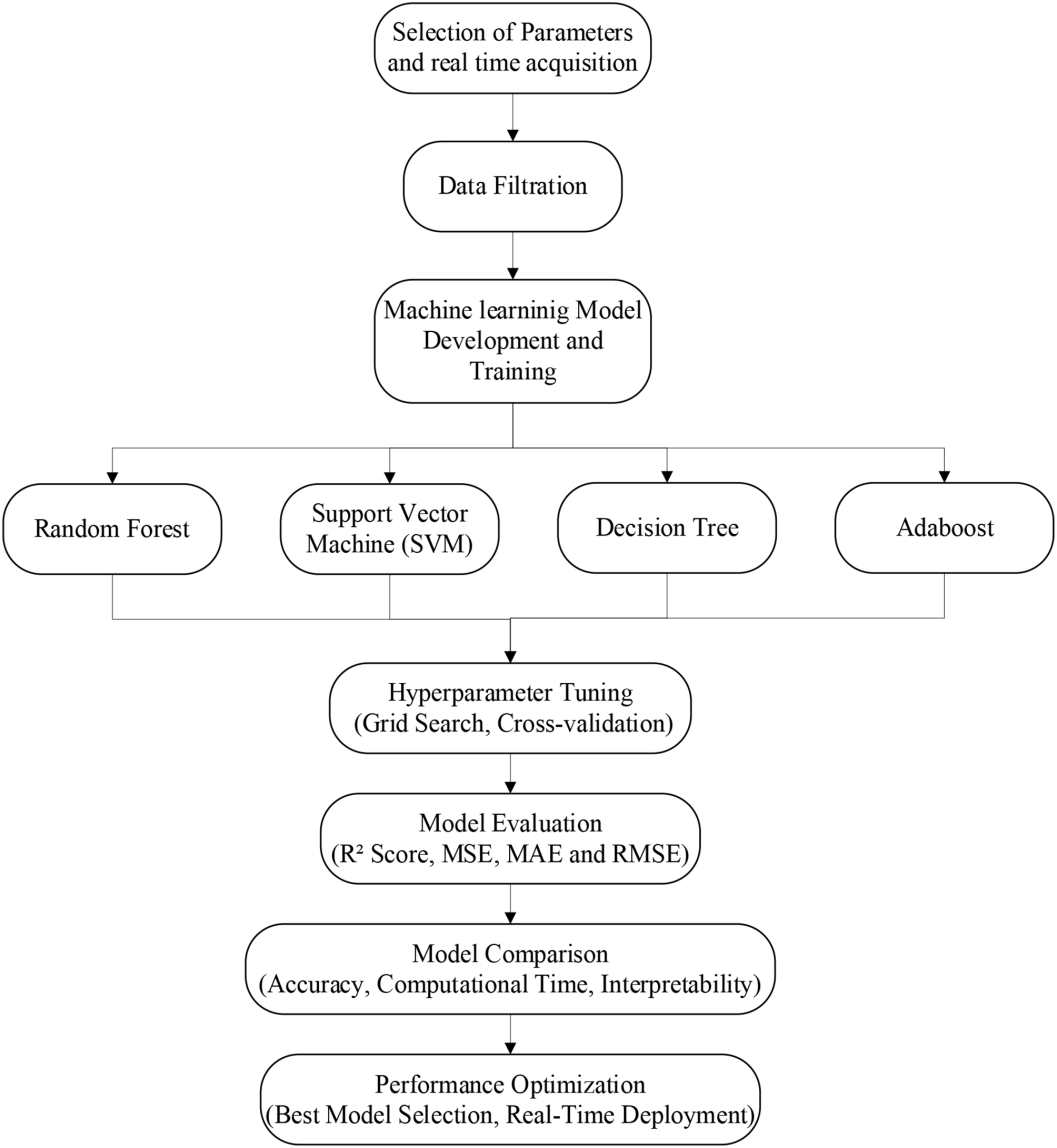
Workflow chart for Cooling Tower Performance Optimization Using Machine Learning Models.

The flowchart of Cooling Tower Performance Optimization Using Machine Learning Models is illustrated in Fig 1. The analysis starts with data collection where real-time and experimental data were collected, including inlet and outlet temperatures, airflow, and water flow rates. Next, data is preprocessed by handling missing data, normalizing features, and choosing relevant variables. Random Forest, Support Vector Machine (SVM), Decision Tree and AdaBoost model were developed in Python using TensorFlow to predict the cooling tower efficiency. An optimization loop is run to enhance the model performance, with a grid search over the hyperparameters and a reduction in the computation process by randomly sampling the hyperparameter values. The performance was evaluated using the Root Mean Squared Error (RMSE) and the Mean Absolute Percentage Error (MAPE) and the accuracy, computational efficiency and interpretability were compared with a comparative analysis. The best model was then chosen for real time deployment to optimize cooling tower operational strategies.

### 2.2. Machine learning models

For the optimization of the cooling tower performance, four machine learning models were used in this study. An overview of each model and how it has been applied in this context is given below.

#### 2.2.1. Random forest.

In the application of the cooling tower study the Random Forest has been employed to predict the high dimensional data and achieve higher predictive accuracy. It is a technique that creates several decision trees using bootstrapped samples with the help of random feature choice to avoid overfitting and, at the same time, keeps the prediction accuracy high. Random Forest was indeed tested and used in the context of HVAC systems, such as cooling towers, to the fault detection and diagnosis problem, thus contributing to improving the efficiency of detecting anomalies and maximizing system performance [37]. However, the disadvantage of this model is that it is computationally more complex than simpler models and less interpretable [38].

#### 2.2.2. Support vector machine (SVM).

The supervised learning is used to determine the optimum hyperplane in the SVM model separating the data for classification and/or regression. They come up with a better generalization by mapping it out, meaning defining actual data points that determine the hyperplane and define the margin between the classes. SVMs have been applied to predict fouling in thermal power plant condensers in cooling systems as part of the effort to aid in maintenance and optimize efficiency. Furthermore, SVMs were employed for condition monitoring and fault analysis of reactor coolant pumps in the nuclear power plants to increase their safety and reliability. SVMs are attractive because they perform well in high-dimensional spaces and are resistant to overfitting in situations when the boundary separation is distinct and obvious. Disadvantages include the need for large computational resources, in particular, when dealing with a large dataset, but challenges in the selection of kernel function [39].

#### 2.2.3. Decision tree.

Supervised learning models decision trees are used to find out the solution, which leads to classification and regression tasks by recursively splitting over the dataset to create a treefish structure of the decision rule. Decision Trees have been used to design a model for the aeration effectiveness of circular plunging jets to optimize aeration processes in cooling systems [40]. The advantages of Decision Trees are that they are simple to deal with (whether they are numerical or categorical data) and they are also easy to interpret. The disadvantages are that it can overfit, particularly for the more complex trees, and is sensitive to details in the data, leading to instability [41–43].

#### 2.2.4. AdaBoost.

The AdaBoost is a popular boosting method that is known to improve a set of weak classifiers into a strong one. It is therefore very successful in applications such as the cooling tower and uses an iterative method to adjust weights to attend to misclassified situations. In cooling tower systems, AdaBoost has been applied to optimize energy efficiency and to forecast maintenance requirements, which ultimately leads to lower operation expenses and higher reliability [44,45]. The algorithm is adaptable to different data types and can be used to enhance the accuracy of the classification. However, challenges such as sensitivity to noisy data and over-fitting remain. Despite the above drawbacks, use of AdaBoost is still useful in industry, such as in cooling towers, to improve system performance.

### 2.3. Model training and development

In the present work, the SVM models were developed to estimate the important parameters of cooling tower, such as outlet water temperature, effectiveness and second law efficiency. The choice of different kernel functions and hyperparameters was done with the aim of getting the best prediction accuracy.

Most of the variables were modeled with Radial Basis Function (RBF) kernel because it can model a nonlinear relationship, whereas a linear kernel was employed in the case of the blowdown loss that has a comparatively linear behavior. The hyperparameters of all the models, including SVM, Random Forest, Decision Tree, and AdaBoost, were tuned using a grid search in conjunction with 5-fold cross validation for optimal performance. Wide range of search space for the key parameters for the SVM like penalty factor (C) and kernel coefficient, and for the ensemble models like tree depth and estimator count. To ensure strong generalisation and avoid overfitting, the best configuration for every algorithm was chosen based on the lowest RMSE. The hyperparameter C = 1000 was selected for the SVM models following a comprehensive Grid Search and cross-validation procedure. During the optimization phase, a range of C values (e.g., 0.1, 1, 10, 100, 1000) was evaluated. It was observed that lower values of C (high regularization) resulted in underfitting, failing to capture the non-linear sensitivity of the second law efficiency to changes in ambient temperature. Conversely, C = 1000 provided the optimal balance between predictive precision and generalization. The epsilon (ε) values were adjusted depending on the sensitivity of the predicted variable, with smaller values used for loss parameters to improve prediction precision. Table 2 summarizes the kernel functions and optimized hyperparameters that were used in training the model.

**Table 2:** Parameters of Random Forest, SVM, Decision Tree, and AdaBoost Model.

Variable	Kernel function	C	Epsilon
Outlet Water Temperature	rbf	1000	0.1
Wet Bulb Temperature	rbf	1000	0.1
Evaporation Loss	rbf	1000	0.001
Blowdown Loss	Linear	1000	0.001
Makeup Water	rbf	1000	0.1
Effectiveness	rbf	1000	1
Second Law Efficiency	rbf	1000	1

#### 2.3.1. Data handling and validation.

After pre-processing to discard abnormal operating modes (such as start-up, shutdown and sensor failures), 243 operating samples were selected to be used in the data set from January to September 2023. A random train-test split with 80% training and 20% testing was used to obtain a strong test set to evaluate the models. The data leakage problem was prevented by following a strict hold-out validation procedure to maintain the integrity of the predictive models. To prevent any information from the test set from influencing the data model’s fitting, all required data preprocessing (such as feature scaling and data normalisation) was limited to the training set. In addition, only the hyperparameters of ensemble models and SVM were optimized by k-fold cross-validation within training partition, ensuring that the reported MAPE and RMSE values represent true generalization on entirely unseen industrial operational parameters. To ensure physical consistency and capture the inherent interdependencies between weld characteristics, a multi-output modeling approach was adopted. This approach ensures that the inter-dependencies between parameters, such as relationship between exit water temperature, effectiveness, and second law efficiency, are captured consistently within the machine learning framework.

### 2.4. Error metrics

#### 2.4.1. Mean squared error (MSE).

It gives a measure of total average size of errors between observed and predicted values, calculated using Eq. 1 [46].


$$\begin{array}{c} MSE\;=\frac{\text{1}}{m}\sum_{i=\text{1}}^{m}(Xi\;-\;Yi{)}^{\text{2}}\; \end{array}$$ (1)

where *X*_*i*_ represents the predicted *i*^*th*^ value, *Y*_*i*_ the actual *i*^*th*^ value and n the number of data points.

#### 2.4.2. Root mean squared error (RMSE).

RMSE is the square root of MSE which is an indicator of error, same units as target variable, facilitating easier interpretation as shown in Eq. 2 [47].


$$\begin{array}{c} RMSE\;=\sqrt{\frac{\text{1}}{m}\sum_{i=\text{1}}^{m}(Xi\;-\;Yi{)}^{\text{2}}} \end{array}$$ (2)

#### 2.4.3. Mean absolute error (MAE).

The average of the absolute discrepancies between the actual and anticipated values is known as Mean Absolute Error. It does not square errors, it gives equal weight to each error, irrespective of the direction. Therefore, it is an advantage to MAE to understand the size of errors rather than to understand if they are overestimated or underestimated. It gives a simple interpretation of the prediction accuracy and can be directly determined by Eq. 3 [47,48].


$$\begin{array}{c} MAE\;=\frac{\text{1}}{m}\sum_{i=\text{1}}^{m}\left|Xi\;-\;Yi\right| \end{array}$$ (3)

where Yi is actual goal value for data set, m represents the number of data points and expected value for data point i is represented by Xi.

#### 2.4.4. R-squared (R²) score.

This is the proportion of variation in dependent variable explained by the model. It is the percentage of variance of the dependent variable that can statistically be explained by the independent variable and it can be calculated by the following Eq. 4 [49].


$$\begin{array}{c} R^{\text{2}}\;=\;\text{1}-\frac{\sum_{i=\text{1}}^{m}(Xi\;-\;Yi{)}^{\text{2}}}{\sum_{i=\text{1}}^{m}{\left(\overset{―}{Y}\;-\;Yi\right)}^{\text{2}}} \end{array}$$ (4)

## 3. Validation

**Fig 2:**
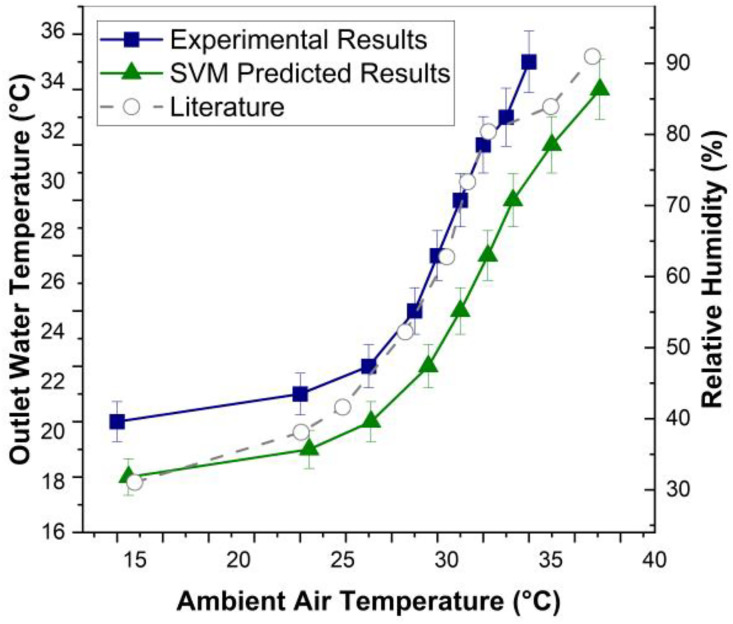
Validation of the machine learning model against experiment and literature results.

The Model results are validated using experimental results, and results presented in the past literature [50]. The results obtained from experiments and past literature for outlet water temperature by changing relative humidity and ambient air temperature show that predicted results are in good agreement, and the model is accurate (see Fig 2).

## 4. Results and discussions

The results of the cooling tower’s performance characteristics are briefly mentioned in the section below.

### 4.1. Performance characteristics

The following discussion will address many aspects of the cooling tower, such as the outlet water temperature, wet bulb temperature, evaporation loss, blow-down loss, makeup water and second-law efficiency.

#### 4.1.1. Impact of humidity and inlet water temperature on outlet water temperature.

**Fig 3:**
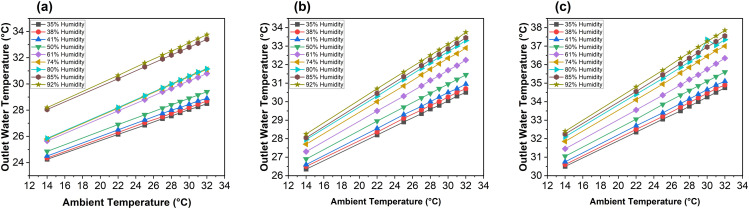
Effect of varying Humidity on Outlet Water Temperature of cooling tower at (a) 32°C, (b) 35°C and (c) 41°C inlet water temperature.

Fig 3 illustrates the variation of outlet water temperature due to the inlet water temperature and humidity. Outlet water temperature decreased due to a decrease in inlet water temperature, ambient air temperature and humidity. A 19.03% increase in outlet water temperature when both humidity and inlet temperature are at their maximum values. On average, humidity alone was found to raise the outlet temperature by 17.87%, 9.51%, and 8.33% corresponding to inlet water temperatures of 32°C, 35°C, and 41°C, respectively.

The ambient air temperature increased due to which the water temperature did not drop to the required limit, resulting in high outlet temperature. The increment in outlet temperature is depicted by the following two reasons, i.e., (1) the higher inlet temperatures, and (2) the humidity content in cooling air increased. Thermal load on tower increases with rise in inlet water temperature [51]. An increase in the moisture content of the air will reduce the potential evaporation, and this will make the heat removal less efficient, resulting in a higher water temperature at the tower’s outlet [52]. Furthermore, increasing relative humidity reduces the cooling effect of evaporation because the air approaches saturation, and there’s less water to be extracted. This results in lower vapour pressure gradient, and thus less evaporation, and hence less heat removal efficiency.

#### 4.1.2. Impact of humidity and inlet water temperature on wet bulb temperature.

**Fig 4:**
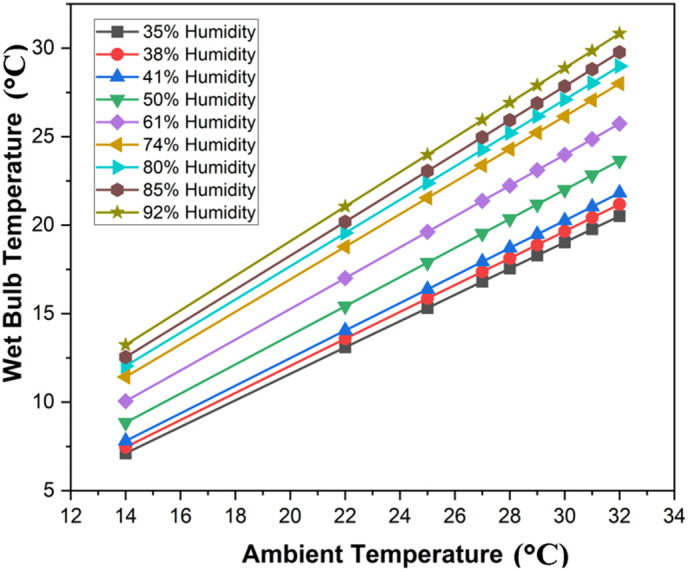
Effect of varying Humidity on Wet Bulb Temperature of cooling tower.

The influence of humidity on wet-bulb temperature is illustrated in Fig. 4, where it can be observed that wet-bulb temperature rises as humidity increases. The wet bulb temperature increased by 86.18% at low ambient air temperatures of 14°C and 50.24% at high ambient air temperatures of 32°C with changing humidity from 35% to 92%. On average wet bulb temperature increased by 57.40% with maximum rise in humidity.

The rise in wet bulb temperature is effective at low ambient temperatures for the inlet air with high humidity content, because of lesser evaporative cooling potential. As relative humidity increases, air’s ability to absorb moisture decreases, resulting in a higher wet bulb temperature even with low ambient temperature [53]. The greater the wet bulb temperatures the lower the cooling capacity of the tower [54]. The greater the level of saturation in the air, the less the difference in the temperatures of the dry bulb and the wet bulb, indicating a reduced capacity of air to absorb moisture through evaporation. The relative effect of humidity is more significant at lower ambient temperatures since the initial moisture content of air in this case is lower and thus a greater relative change in saturation conditions is possible.

#### 4.1.3. Influence of humidity and inlet water temperature on cooling tower water losses.

**Fig 5:**
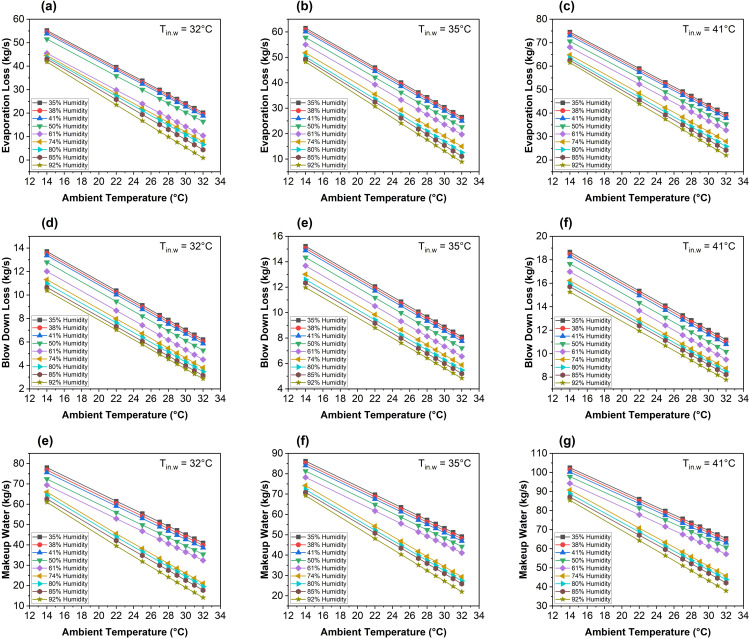
The Effect of ambient humidity and inlet water temperature on (a–c) evaporation loss, (d–f) blowdown loss, and (g–i) makeup water requirement.

The effect on evaporation losses due to humidity and inlet water temperature is studied (see Fig 5a–5c). Evaporation losses were found to reduce as humidity increased, while they increased with higher inlet water temperatures. As humidity increased from 35 to 92% evaporation losses decreased on average by 62.40%. With an increase in inlet water temperature at (a) 32°C, (b) 35°C, and (c) 41°C, evaporation losses increased by 18.08%, 21.96%, and 24.61% respectively.

The rise in evaporation loss is due to the following reasons, (1) decrease in humidity, and (2) increase in inlet water temperature. These trends could be explained in terms of vapor pressure and mass transfer. Water surface and surrounding air vapor pressure difference enhance evaporation. High relative humidity reduces this difference, thereby limiting evaporation [52]. Saturation vapor pressure at the surface of the water increases as the water inlet temperature rise, increasing the driving potential of mass transfer, and therefore increasing evaporation rates.

Fig 5d–5f depicts the variation in blowdown loss under different humidity levels and inlet water temperatures. Like the evaporation loss, blowdown loss also reduces with increasing humidity. The loss in blowdown is reduced by about 55–70% with the rise in humidity of 35–92%, with ambient temperature conditions.

This is due to the fact that at high humidity, evaporation rates decrease, resulting in lowers the rate of dissolved solids in circulating water and thereby minimizing need for blowdown. Conversely, the loss in the blowdown rises as the inlet water temperature rises. The blowdown loss increases by about 18–22% as inlet temperature rises between 32°C and 41°C because evaporation rates are elevated, requiring extra blowdown to restore the water level to satisfactory levels.

Fig 5g–5i indicates the dependence of the makeup water requirement on humidity and the inlet water temperature. The makeup water replenishes the evaporation, blowdown and the small losses of the system. The findings indicate that the requirement of makeup water reduces with the rise in humidity. An increase in humidity from 35% to 92% would result in a reduction of the makeup water demand of about 58% to 68%. As the relative humidity of air increases, the partial pressure of water vapour in the air becomes closer to the saturation vapour pressure of water at the surface and mass transfer is inhibited. This reduces losses due to evaporation which in turn decreases the amount of dissolved solids that accumulate in the circulating water thus reducing the amount of blowdown required. The overall decrease in evaporation and blowdown directly reduces the makeup water demand. Conversely, an increase in inlet water temperature results in a higher makeup water requirement. As the inlet water temperature is raised by 32°C to 35°C and 41°C, the makeup water demand goes up by about 18–25%. This is because the saturation vapor pressure at the water surface increases as the water temperature increases (Clausius-Clapeyron relation). This enhances the evaporation rate. The higher rate of evaporation increases the rate at which the dissolved solids are concentrated and this requires an increase in the rates of the blowdown to ensure that the levels of the dissolved solids remain within acceptable limits.

#### 4.1.4. Impact of humidity and inlet water temperature on second law efficiency.

**Fig 6:**
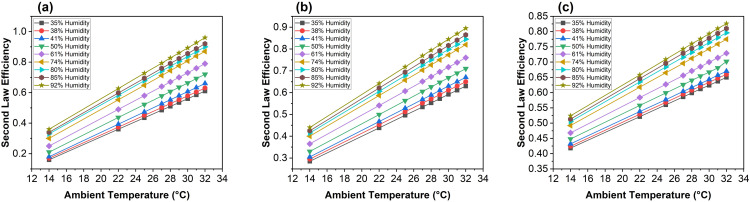
Effect of varying Humidity on Second Law Efficiency of cooling tower at (a) 32°C, (b) 35°C and (c) 41°C inlet water temperature.

Fig 6 de picts the effect of ambient humidity and inlet water temperature on cooling tower efficiency. Results show that, second law efficiency is on the increase with the rise of ambient temperature under all humidity conditions. The second law efficiency is raised by about 65% to 75% with the rise in ambient temperature (14°C to 32°C) and the level of humidity with an inlet water temperature. The decrease in exergy destruction at elevated ambient temperatures can be attributed to this trend. When temperature difference between the system and surroundings becomes smaller, which results in reduced thermodynamic irreversibilities related to heat transfer. As a result, a greater portion of the available exergy is actually used, leading to greater second law efficiency.

The other notable trend observed is that second law efficiency increases with increasing relative humidity. When humidity increases from 35% to 92%, the second law efficiency increases by approximately 35–45% across the studied ambient temperature range. Thermodynamically, increased humidity decreases the rate of evaporation as the difference between in vapor pressures of working fluids decreases. This minimization in the evaporation reduces exergy losses due to phase change and mass transfer to unsaturated air. This leads to a reduction in the irreversibility of the system and an increase in the fraction of the exergy supplied that is useful in providing cooling and so enhancing second law efficiency.

In addition, the second law efficiency is also affected by inlet water temperature. The second law efficiency increases by approximately 12% to 18% with increasing inlet water temperature from 32°C to 41°C. Rise in inlet water temperature enhances heat and mass transfer. It also increases exergy content of incoming stream, enabling a higher recovery of useful work relative to the exergy input, ultimately leading to improved second-law efficiency.

Generally, the findings show that ambient temperature, humidity, and inlet water temperature increase is linked with second law efficiency. Among these parameters, humidity shows a strong influence, which increases efficiency by up to 45% when humidity increases from 35% to 92%. The results indicate that environmental conditions are significant determinants of thermodynamic performance of cooling tower systems.

### 4.2. Forecasting of cooling tower performance parameters

**Fig 7:**
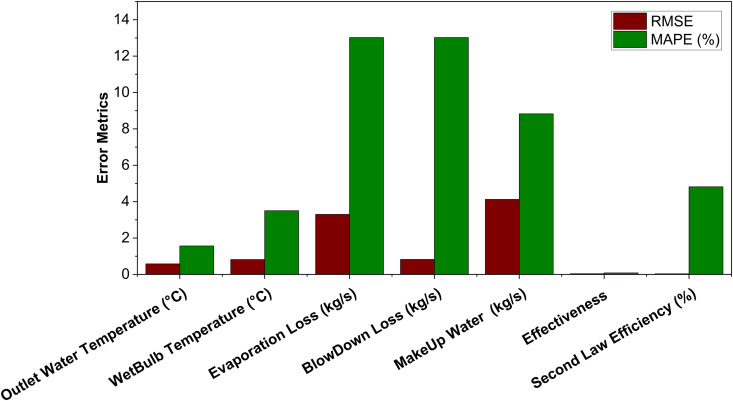
Performance evaluation of the Adaboost model using various metrics.

The evaluation of the Adaboost model, presented in Fig 7, reveals strong predictive capabilities across all cooling tower performance parameters. The model attained MAPE values of 3.5%, 13.02%, 1.56%, 13.02%, 8.82%, 0.08%, and 4.82% for temperature of the wet bulb, evaporation loss, and exit water, blowdown loss, makeup water, effectiveness, and second law efficiency, respectively. These low error margins demonstrate high prediction accuracy and minimal deviation from experimental results. Furthermore, the RMSE values remain comparatively small across all parameters highlighting the model’s precision and stability under varying operating conditions. Model achieve overall R² of 0.973. Lowest RMSE values was observed for second law efficiency of 0.03%. Despite its robustness, minor fluctuations in certain parameters such as evaporation and blowdown losses suggest that ensemble boosting may still be sensitive to slight data inconsistencies. Nevertheless, Adaboost is a highly efficient algorithm, suitable for reliable and accurate predictive modeling in cooling tower performance forecasting.

**Fig 8:**
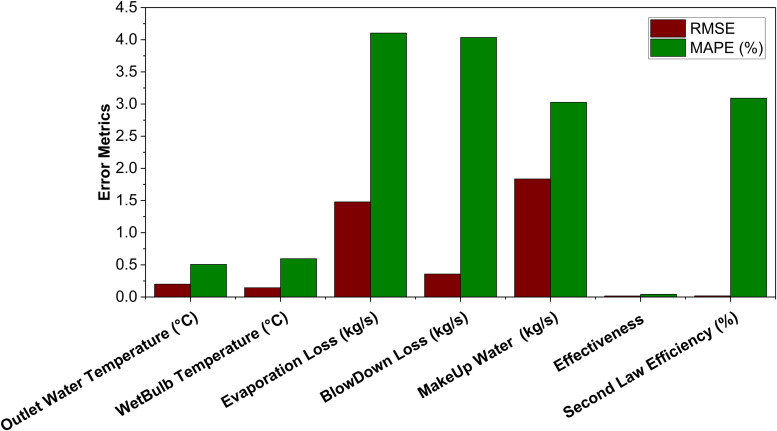
Performance evaluation of the Random Forest model using various metrics.

The Random Forest model exhibits strong forecasting ability for cooling tower parameters, although with some variability across parameters (see Fig 8). It achieved MAPE values of 3.8%, 12.4%, 1.16%, 12.39%, 8.9%, 0.12%, and 4.4% for temperature of the wet bulb, evaporation loss, and exit water, blowdown loss, makeup water, effectiveness, and second law efficiency, respectively. While the model shows excellent predictive accuracy for parameters such as effectiveness and outlet temperature, the relatively higher errors observed in evaporation loss, blowdown loss, and makeup water predictions indicate limitations in handling specific thermal losses. Nevertheless, the model maintains low RMSE values of 0.02% for second law efficiency and maximum of 1.83 kg/s for makeup water and R² value of 0.9801, reflecting strong general stability and resilience against overfitting. Random Forest is particularly robust for modeling complex thermodynamic behaviors, yet its performance varies with parameters exhibiting high variability or nonlinearities.

**Fig 9:**
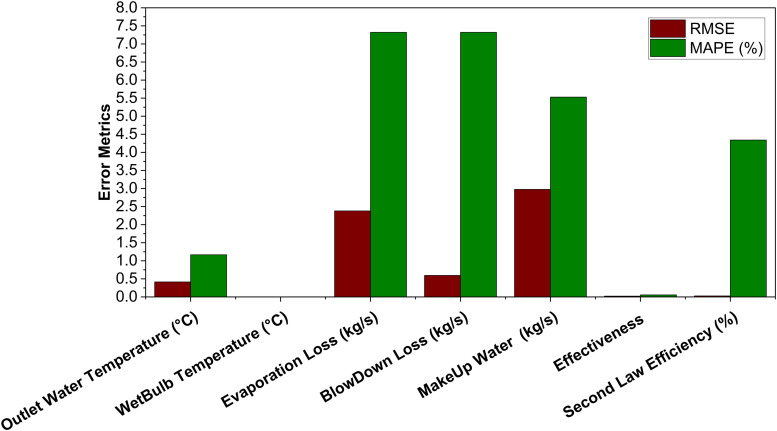
Performance evaluation of the decision tree model using various metrics.

The Decision Tree model’s forecasting results, depicted in Fig 9, demonstrate moderate predictive accuracy across the analyzed parameters. The model achieved MAPE values of 0.01%, 7.32%, 7.32%, 5.52%, 0.05%, and 4.34% for temperature of the wet bulb, evaporation loss, and exit water, blowdown loss, makeup water, effectiveness, and second law efficiency, respectively. Although satisfactory for simpler parameters, the Decision Tree model exhibits higher error margins for parameters associated with evaporation and blowdown losses, achieving an overall R² value of 0.971. Moreover, RMSE values indicate increased sensitivity to data noise and overfitting compared to ensemble methods. The simplicity and interpretability of the Decision Tree model are advantageous for quick assessments and preliminary studies; however, its comparatively lower predictive strength renders it less suitable for high-precision forecasting where dynamic and nonlinear behaviors dominate.

**Fig 10:**
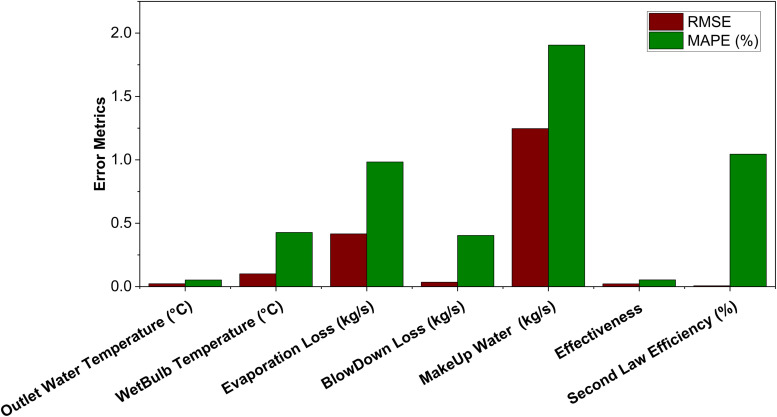
Performance evaluation of the SVM model using various metrics.

The SVM model’s forecasting performance, shown in Fig 10, significantly outperforms in terms of prediction and stability. Impressively low MAPE values of 0.42%, 0.98%, 0.05%, 0.40%, 1.91%, 0.05%, and 1.04% were obtained for temperature of the wet bulb, evaporation loss, and exit water, blowdown loss, makeup water, effectiveness, and second law efficiency, respectively. Additionally, the RMSE values approaches maximum of 1.25 kg/s for makeup water and lowest of 0.01% for second law efficiency further validates the model’s precision and robustness across all operational parameters. The overall R^2^ value of 0.985 is achieved. SVM’s consistency in maintaining minimal reduction errors across diverse variables highlights its superior generalization capability, making it the most suitable model for accurate and reliable forecasting in cooling tower applications.

## 5. Conclusion

This study introduced a universal machine learning-driven model for the prediction and analysis of cooling towers in terms of their thermodynamic and operational performance. The four machine learning algorithms were tested based on operational parameters such as inlet water temperature, ambient air temperature and relative humidity to predict the key performance indicators such as outlet water temperature, evaporation loss, blowdown loss, makeup water requirement, effectiveness and second-law efficiency.

- Based on the evaluation results, the Support Vector Machine (SVM) model emerges as the most accurate and reliable model for forecasting cooling tower performance parameters. The variance of the prediction errors is represented by the reported statistical metrics, specifically the RMSE and MAPE. For best-performing SVM model, the maximum RMSE was 1.25 kg/s, and MAPE was 1.89%, indicating a very low dispersion of errors. This indicates that model predictions remain in close agreement with the experimental data over the full range of operating conditions.
- AdaBoost also demonstrated good performance with most prediction errors falling below 4%, whereas the performance of the Random Forest was moderate, with deviations in evaporation and blowdown losses, which were higher (MAPE up to about 12%). The Decision Tree model, which was simple and interpretable, demonstrated relatively low accuracy because it is sensitive to data variation.
- Parametric analysis indicates that increasing relative humidity from 35% to 92% reduced evaporation and blowdown losses by 55–70%, decreasing makeup water demand by 58–68%. At the same time, wet-bulb temperature increased significantly, with an average rise of approximately 57% across the studied range, indicating reduced evaporative cooling potential at higher humidity levels.
- Increase in inlet water temperature (32–41°C) increased the evaporation losses, up to 24.6% and the blowdown losses and makeup water demand increased by 18–25%.
- Additionally, outlet water temperature increased by up to 19% under combined high humidity and high inlet temperature conditions, which indicated the low cooling efficiency in unfavorable environmental conditions.
- Thermodynamic results showed that second-law efficiency improved by 65–75% with increasing ambient temperature, 35–45% with higher humidity, and 12–18% with elevated inlet water temperature.

The study demonstrates that machine learning algorithms are capable of accurately capturing complicated nonlinear interactions between environmental factors and cooling tower performance from a qualitative standpoint. While the present study focuses on deterministic machine learning models with strong predictive performance, incorporating uncertainty quantification techniques, such as Bayesian methods or Gaussian Process models, is recommended for future work. The enhancements to the predictive framework can guide industrial operators to improve cooling efficiency, reduce water use, and manage sustainable energy in thermal systems.

### Nomenclature

**Table:** 

T_amb_	Ambient air temperature (K)
T_in,w_	Inlet water temperature (K)
T_out,w_	Outlet water temperature (K)
R^2^	Coefficient of Determination
T_out,w_	Outlet water temperature (K)
Φ	Relative humidity (%)
ṁ_w_	Mass flow rate of water (kg/s)
ṁ_a_	Mass flow rate of air (kg/s)
EL	Evaporation loss
BL	Blowdown loss
η	Effectiveness
η_II_	Second law efficiency
